# GCKSign: Simple and efficient signatures from generalized compact knapsack problems

**DOI:** 10.1371/journal.pone.0310708

**Published:** 2024-09-23

**Authors:** Joo Woo, Kwangsu Lee, Jong Hwan Park

**Affiliations:** 1 Graduate School of Information Security, Korea University, Seoul, South Korea; 2 Department of Computer and Information Security, Sejong University, Seoul, South Korea; 3 Department of Computer Science, Sangmyung University, Seoul, South Korea; Deakin University, AUSTRALIA

## Abstract

In 2009, Lyubashevsky proposed a lattice-based signature scheme using the Schnorr-like identification and the Fiat-Shamir heuristic and proved its security under the collision resistance of a generalized compact knapsack function. However, their security analysis requires the witness indistinguishability property, leading to significant inefficiency and an increase of sizes of public key and signature. To overcome the efficiency issue associated with the WI property, we introduce a new lattice-based assumption, called the target-modified one-wayness problem of the GCK function and show its reduction to well-known lattice-based problems. Additionally, we present a simple and efficient GCK-based signature scheme, GCKSign, whose security is based on the Module GCK-TMO problem in the random oracle model. GCKSign is a natural extension of Lyubashevsky’s scheme in a module setting, but achieves considerable efficiency gains due to eliminating the witness indistinguishability property. As a result, GCKSign achieves approximately 3.4 times shorter signature size and 2.4 times shorter public key size at the same security level.

## 1 Introduction

The generalized compact knapsack (GCK) function [1] is defined with a ring-Short Integer Solution (SIS) instance over a polynomial-based ring *R*_*n*,*q*_ for some modulus *q*. Specifically, for a random ring-SIS instance $a=\left(a_{1},\ldots ,a_{m}\right)\in R_{n,q}^{m}$, the GCK function $F_{a}:R_{n,q}^{m}\to R_{n,q}$ is computed as $t=F_{a}\left(x\right)=\sum_{i=1}^{m}a_{i}x_{i}$ for a domain element $x\in R_{n,q}^{m}$ with short coefficients. In 2002, Micciancio [1] showed that the function *F*_***a***_ is one-way, assuming the worst-case hardness of some shortest independent vector problems (SIVP) on cyclic lattices in a ring. Furthermore, in 2006, researchers [2, 3] proved that the GCK function *F*_***a***_ is collision-resistant, assuming the worst-case hardness of some shortest vector problems (SVP) for ideal lattices in a ring. Additionally, the GCK function has the linearity property, which states that *F*_***a***_(*c*_1_***x***_1_ + *c*_2_***x***_2_) = *c*_1_*F*_***a***_(***x***_1_) + *c*_2_*F*_***a***_(***x***_2_) for any ring elements *c*_1_, *c*_2_ ∈ *R*_*n*,*q*_ and any domain elements $x_{1},x_{2}\in R_{n,q}^{m}$.

Based on the aforementioned properties of the GCK function, Lyubashevsky [4] proposed a GCK-based signature scheme in 2009, using the Schnorr-like identification protocol and the Fiat-Shamir transform [5]. The main idea of [4] is to set up a public key as (***a***, *t* = *F*_***a***_(***s***)) and its corresponding signing key as ***s***, where $s\in R_{q}^{m}$ is a vector of short polynomials. The signing procedure begins by sampling a short vector $y\in R_{q}^{m}$ (from a suitable distribution) and computing *v* = ***ay***(= *F*_***a***_(***y***)). The signer then computes *c* = *H*(*v*, *μ*), where *H* is a hash function and *μ* is a message, and ***z*** = ***y*** + ***s****c*. To ensure that the distribution of ***z*** is independent of the secret ***s***, preventing any leakage of information about ***s***, the signer employs a rejection rule on ***z***. Following this *rejection sampling*, a signature (***z***, *c*) is generated only when ***z*** is sampled from the (predefined ***z***’s) distribution centered at zero, rather than at ***s****c*. Subsequently, the verifier checks whether *H*(***az*** − ***t****c*, *μ*) is equal to *c*, and ***z*** is sufficiently small.

Although the GCK-based signature proposed by Lyubashevsky has a similar structure with the previous Schnorr signature, their security proof employed in [4] relies on the concept of *witness indistinguishability* (WI). This notion is essential for proving the security of [4] under the assumption of collusion resistance of the GCK function. WI refers to the property that when choosing an alternative (or multiple) signing key ***s***′ such that ***t*** = *F*_***a***_(***s***) = *F*_***a***_(***s***′), the signature (***z*** = ***y*** + ***s****c*, *c*) is (statistically) indistinguishable from (***z*** = ***y*** + ***s***′*c*, *c*). In the security analysis, all signatures are simulated using ***s***, but due to the WI property, the adversary cannot know the exact secret key ***s***. Thus, with high probability, it is expected that the adversary succeeds in forging a signature with the other ***s***′. In such a case, the rewinding technique [6] enables a reductionist to extract a pair of distinct inputs as a collision of *F*_***a***_. Since breaking the collision resistance of the GCK function implies the ability to solve a ring-SIS problem, the security of the GCK-based signature scheme [4] eventually relies on the hardness of a ring-SIS problem.

However, the WI property results in a significant efficiency drawback in [4]. This stems from the fact that the public key *t* = *F*_***a***_(***s***) and the corresponding secret key ***s*** are generated in such a way as to guarantee collisions in *F*_***a***_. Specifically, ***s*** needs to be parametrized so that there exists another valid secret key ***s***′ such that *F*_***a***_(***s***) = *F*_***a***_(***s***′). This requirement mandates that each coefficient of the secret key ***s*** be sampled from a relatively large range, for example, [−2047, 2047], resulting in large size of the signature (***z***, *c*). To overcome the efficiency issue associated with the WI property and the large bound on ***s***, Lyubashevsky [7] presented an alternative proof technique based on the *decisional* ring-SIS problem, where *t* = *F*_***a***_(***s***) with a small bound on ***s*** is indistinguishable from a uniformly random *t* in *R*_*q*_. Consequently, a (real) GCK-based signature scheme is constructed using ***s*** with small coefficients, while a simulated signature scheme in the security proof is provided to the adversary using ***s***′ with large coefficients. Although the WI property with a small bound on ***s*** enables a more efficient GCK-based signature scheme, no known quantum reduction exists from worst-case lattice problems to the decisional ring-SIS problem in ideal lattices [7].

Another approach to circumvent the WI property with large coefficients of ***s*** is to construct the public key *t* using the Learning with Errors (LWE) problem. In this approach, *t* is computed as *t* = *F*_***a***_(***s***) + *e*, where *e* is a polynomial sampled from a narrow distribution. Based on the decisional ring-LWE problem, which is to distinguish between a ring-LWE instance and a uniformly random one, the security proof of a ring-LWE-based signature scheme can be established using a similar argument as that of the decisional ring-SIS problem. Importantly, this approach provides improved security against quantum adversaries because there is a reduction from worst-case lattice problems to the decisional ring-LWE problems [8]. Leveraging this idea, Güneysu et al. [9] proposed a ring-LWE-based signature scheme where a signature consists of three components (*c*, ***z***_1_, *z*_2_), where ***z***_1_ = ***y***_1_ + *c****s*** and *z*_2_ = *y*_2_ + *ce*. Bai and Galbraith [10] reduced the signature size of [9] by omitting *z*_2_ and introducing a *compression* technique to compensate for the correctness error resulted from *e*. In 2018, Ducas et al. [11] proposed a signature scheme called Dilithium, which can be viewed as a generalization of [10] based on Module-LWE problems. Additionally, [11] presented a distinct proof technique that does not rely on the WI property used previously. They employed two signature forgeries (by rewinding an adversary) to directly solve a ring-SIS problem concerning (***A***||***t***||1) rather than (***A***||1). Many other signatures based on [4] were proposed [12, 13] recently and their security proof also follow the way of Dilithium.

### 1.1 Our contribution

From what has been shown so far, while significant progress has been made in creating efficient lattice-based signatures, the following question still arises:

*Can we prove the security of GCK-based signature without using the WI property*?

The goal of this paper is to give a positive answer to the above problem. To achieve this, we define a relaxed version of the one-wayness problem of the GCK function, called the target-modified one-wayness (TMO) problem. In essence, the TMO problem is to solve the one-wayness problem of the GCK function *approximately* rather than exactly: given $a=\left(a_{1},\ldots ,a_{m}\right)\in R_{n,q}^{m}$ and ***t*** = *F*_***a***_(***s***) ∈ *R*_*n*,*q*_ for some $s\in R_{n,q}^{m}$, find short polynomials $\left(x=\left(x_{1},\ldots ,x_{m}\right),c\right)\in R_{n,q}^{m}\times R_{q}$ such that *F*_***a***_(***x***) = *c****t***. To provide confidence in the TMO problem, we show that the TMO problem is reduced to both the one-wayness and the collision-resistance problems of the GCK function.

Instead of proving the original GCK-based signature scheme [4], we present a more efficient GCK-based signature scheme called *GCKSign*, which is a natural extension of [4] to the Module-GCK function. We adopt a matrix form of $A\in R_{q}^{k\times \ell }$ and $s\in R_{q}^{\ell \times 1}$ and define a Module-GCK function as $t=F_{A}\left(s\right)=As\in R_{q}^{k\times 1}$. Similar to the TMO problem, we establish a Module GCK-TMO problem, and show its reduction to Module-SIS, LWE problems. Notably, we prove that GCKSign is secure in the random oracle model under the Module GCK-TMO problem without relying on the WI property. By eliminating this property, we achieve significant efficiency improvements. In detail, GCKSign achieves a signature size that is about 3.4 times shorter and a public-key size that is about 2.4 times shorter at the same security level, compared to [4]. In Section 5, we provide a concrete security and efficiency comparison between [4] and GCKSign.

## 2 Preliminaries

We begin by defining the syntax and security of digital signature. We also define two computational hardness problems related to the GCK function.

### 2.1 Notation

For a modulus q∈N, $Z_{q}$ denotes a quotient group with respect to addition modulo *q*. Let *R*_*n*_ and *R*_*n*,*q*_ respectively be the rings $Z\left[x\right]/\left(x^{n}+1\right)$ and $Z_{q}\left[x\right]/\left(x^{n}+1\right)$, where *n* is a power of two. Vectors with entries in *R*_*n*,*q*_ are denoted by bold lowercase letters, for example, $a=\left(a_{1},\ldots ,a_{m}\right)\in R_{n,q}^{m}$ where *a*_1_, …, *a*_*m*_ ∈ *R*_*n*,*q*_ for some positive integer *m*. $R_{n,q}^{\left(h\right)}$ denotes a subset of *R*_*n*,*q*_, consisting of polynomials with coefficients of which only a fixed number of *h* is −1 and 1, and all other coefficients are zero. We notice that |Rn,q(h)|=2h×(nh). For a positive integer *x*, *R*_[−*x*,*x*]_ denotes a set of *R*_*n*,*q*_, consisting of polynomials with coefficients between [−*x*, *x*]. The notation ‖⋅‖_∞_ refers to the infinity norm.

### 2.2 Digital signature

**Definition 1**. *A digital signature (DS) scheme for a message space* M *consists of three algorithms*: KeyGen, Sign, *and* Verify *such that*:

- *KeyGen*(λ): *The key generation algorithm takes as input a security parameter* λ *and outputs a pair of keys* (*pk*, *sk*). *These keys are called the public key and the private key*.
- *Sign*(*sk*, *μ*): *The signing algorithm takes as input the private key sk and a message* μ∈M, *and then outputs a signature σ*.
- *Verify*(*pk*, *μ*, *σ*): *The verification algorithm takes as input the public key pk, a message μ and a signature σ, and then outputs 1 if the signature is valid or 0 otherwise*.

**Definition 2** (Existential unforgeability). *Let* DS=(KeyGen,Sign,Verify) *be a digital signature scheme. The existential unforgeability against chosen-message attacks (UF-CMA) is defined via the following experiment* ${UF-CMA}_{DS}^{A}(\lambda )$ *between a challenger* C *and an adversary* A:

1.
C *runs the key generation algorithm to get* (*pk*, *sk*) *and gives pk to* A.
2.
A *queries a signing oracle with a message μ*. *Let* Q *denote the set of all queries that* A *queried*.
3. *Finally*, A outputs a signature σ* and a message μ*. C *returns 1 if (1) Verify*(*pk*, μ*, σ*) = 1 *and (2)* $\mu^{*}\notin Q$, *and otherwise returns 0 as the output of the game*.

*The advantage of* A *for breaking the UF-CMA security of DS is defined as* ${\text{Adv}}_{DS}^{UF-CMA}\left(A\right)=\text{Pr}\left[UF-{CMA}_{DS}^{A}\Rightarrow 1\right]$ . *We say that a DS scheme is UF-CMA secure if for any polynomial-time adversary* A, *we have* ${\text{Adv}}_{DS}^{UF-CMA}\left(A\right)\leq \epsilon \left(\lambda \right)$, *where ϵ is a negligible function for the security parameter* λ.

### 2.3 GCK hardness problems

**Definition 3** (GCK Function [1]). *For a ring R*_*n*,*q*_, *a subset S* ⊂ *R*_*n*,*q*_, *an integer m* ≥ 1, *and a randomly and independently chosen* $a=\left(a_{1},\ldots ,a_{m}\right)\in R_{n,q}^{m}$, *the GCK function F*_***a***_: *S*^*m*^ → *R*_*n*,*q*_ *is defined as follows*:

$$\begin{array}{c} F_{a}\left(x\right)=\sum_{i=1}^{m}a_{i}x_{i} \end{array}$$ (1)

*for* $x\in S^{m}\subset R_{n,q}^{m}$, *where* $\sum_{i=1}^{m}a_{i}x_{i}$ *is computed using the ring multiplication and addition operations*.

In this paper, we specify the subset *S* as *S* = *R*_[−*β*,*β*]_ for some integer *β*.

**Definition 4** (One-Wayness of GCK function [1]). *A GCK function is one-way (OW) if for any probabilistic polynomial-time (PPT) algorithm* A, *it is easy to compute, but computationally hard to invert the GCK function: given a pair* (***a***, *t* = *F*_***a***_(***x***)) *for a randomly chosen* $a\in R_{q}^{m}$ *and* $x\in R_{\left[-\beta ,\beta \right]}^{m}$, *find* ***x*** *in the domain such that* *F*_***a***_(***x***) = *t*. *For integers* n,m,q,β∈N, *we define* ${\text{Adv}}_{n,m,q,\beta }^{OW}$ *to be the advantage of an algorithm* A *in solving the OW problem of a GCK function over the ring R*_*n*,*q*_.

**Definition 5** (Collision-Resistance of GCK function [2, 3]). *A GCK function is collision-resistant (CR) if for any probabilistic polynomial-time (PPT) algorithm* A, *it is computationally hard to find a collision of a GCK function: given a randomly chosen* $a\in R_{q}^{m}$, *find distinct* $x,x^{\prime }\in R_{\left[-\beta ,\beta \right]}^{m}$ *such that F*_***a***_(***x***) = *F*_***a***_(***x***′). *For integers* n,m,q,β∈N, *we define* ${\text{Adv}}_{n,m,q,\beta }^{CR}$ *to be the advantage of an algorithm* A *in solving the CR problem of a GCK function over the ring R*_*n*,*q*_.

## 3 Main results

### 3.1 GCK-TMO problem

Now, we define a new GCK-related problem, called TMO problem of the GCK function.

**Definition 6** (Target-Modified One-wayness of GCK function). *For integers* n,m,q,α,β∈N, *the TMO problem is defined as follows: given* $a\in R_{n,q}^{m}$ *and t* ∈ *R*_*n*,*q*_, *find* $x\in R_{n,q}^{m}$ *and c* ∈ *R*_*n*,*q*_ *such that* ‖*c*‖_∞_ ≤ *α*, ‖***x***‖_∞_ ≤ *β satisfying*

$$\begin{array}{c} F_{a}\left(x\right)=ct. \end{array}$$ (2)

The TMO problem is a modified OW problem of a GCK function, obtained by changing the original target *t* into a new *ct*. The important point is that *c* ∈ *R*_*q*_ should be short, meaning that ‖*c*‖_∞_ ≤ *α* for a small integer *α*, and can also be chosen by a solver as desired. Intuitively, the TMO problem becomes trivial if *c* is chosen freely in *R*_*q*_, because for a short ***x*** such that ‖***x***‖_∞_ ≤ *β*, *F*_***a***_(***x***) = *t*′ is firstly computed and then *c* is obtained via *c* = *t*′*t*^−1^ (if *t*^−1^ exists). Also, the OW problem of a GCK function can be viewed as a special case of the TMO problem by issuing (***x***, *c* = 1) as a solution. For integers n,m,q,α,β∈N, we define ${\text{Adv}}_{n,m,q,\alpha ,\beta }^{\text{TMO}}$ to be the advantage of an algorithm A solving the TMO problem over the ring *R*_*n*,*q*_.

#### 3.1.1 Computational hardness of the TMO problem

By deriving an upper bound on ${\text{Adv}}_{n,m,q,\alpha ,\beta }^{\text{TMO}}$ from the following reduction, we demonstrate that the TMO problem is at least as hard as the CR and OW problems of a GCK function. For our reduction, we require a special form of (*n*, *q*) that determines a ring $R_{q}=Z_{q}/\left(x^{n}+1\right)$, in order to guarantee that a short *c* ∈ *R*_*q*_ has an inverse. More precisely, we require that *n* and *ρ* are power-of-2 integers such that *n* ≥ *ρ*, and *q* is a prime such that *q* ≡ 2*ρ* + 1 (mod 4*ρ*). We then use the result [14, Corollary 1.2.] that, for any short *c* ∈ *R*_*q*_ such that ${\left\|c\right\|}_{\infty }<\left(1/\sqrt{\rho }\right)\times q^{1/\rho }$, *c* has an inverse in *R*_*q*_ with probability 1.

**Theorem 1**. *Let n and ρ be power-of-2 integers such that n* ≥ *ρ*, *and q is a prime such that q* ≡ 2*ρ* + 1 (mod 4*ρ*). *For integers* m,α,β,γ∈N *satisfying* $\alpha <\left(1/\sqrt{\rho }\right)\times q^{1/\rho }$, (2*β* + 1)^*mn*^ ≫ *q*^*n*^ *and nαγ* ≤ *β*, *it holds that* ${\text{Adv}}_{n,m,q,\alpha ,\beta }^{TMO}\leq {\text{Adv}}_{n,m,q,\beta }^{CR}+{\text{Adv}}_{n,m,q,\gamma }^{OW}$.

*Proof*. Suppose there is an algorithm A that solves the TMO problem with advantage ${\text{Adv}}_{n,m,q,\alpha ,\beta }^{\text{TMO}}$ for any *α* such that $\alpha <\left(1/\sqrt{\rho }\right)\times q^{1/\rho }$. Recall that A takes (***a***, *t*) as input and tries to find a pair (***x***, *c*) such that *F*_***a***_(***x***) = *ct*, satisfying the condition that ‖*c*‖_∞_ ≤ *α*, ‖***x***‖_∞_ ≤ *β*. According to [14, Corollary 1.2.], any *c* ∈ *R*_*n*,*q*_ that satisfies ${\left\|c\right\|}_{\infty }<\left(1/\sqrt{\rho }\right)\times q^{1/\rho }$ has an inverse in *R*_*n*,*q*_. Since *α* is less than $\left(1/\sqrt{\rho }\right)\times q^{1/\rho }$, the short polynomial *c* always has an inverse in *R*_*n*,*q*_. With (***x***, *c*) that A outputs, we set ***z*** = ***x****c*^−1^ by considering *c*^−1^ as a scalar. Since the GCK function *F*_***a***_ is linear, we see that *F*_***a***_(***z***) = *F*_***a***_(***x****c*^−1^) = *c*^−1^*F*_***a***_(***x***) = *c*^−1^*ct* = *t*. For the integer γ∈N satisfying *γ* ≤ *β*/*nα*, we consider two cases as follows:

**Case 1**: ‖***x****c*^−1^‖_∞_ > *γ*.

**Case 2**: ‖***x****c*^−1^‖_∞_ ≤ *γ*.

Obviously, A’s output (***x***, *c*) belongs to either case 1 or case 2. In case 1, we show that an algorithm B uses A to solve the CR problem of a GCK function. The assumption that (2*β* + 1)^*mn*^ ≫ *q*^*n*^ guarantees that it is presumably feasible to find a pair of collision with respect to any element in *R*_*q*_. Given $a\in R_{q}^{m}$ as input, B does as follows:

1. Choose a random $z^{\prime }\in R_{\left[-\gamma ,\gamma \right]}^{m}$.
2. Compute $t=F_{a}\left(z^{\prime }\right)$.
3. Run A on input (***a***, *t*) and get (***x***, *c*) from A.
4. Compute ***x***′ = *c****z***′ and output (***x***, ***x***′) as a solution.

For (***x***, ***x***′) to be a solution of the CR problem, we need to show that *F*_***a***_(***x***) = *F*_***a***_(***x***′), ‖***x***‖_∞_ ≤ *β*, ‖***x***′‖_∞_ ≤ *β*, and ***x*** ≠ ***x***′. First, since (***x***, *c*) is a solution for the TMO problem, it means that *F*_***a***_(***x***) = *ct*. Also, we set *t* = *F*_***a***_(***z***′) in step 2. By the linearity property of GCK function, it holds that *ct* = *cF*_***a***_(***z***′) = *F*_***a***_(*c****z***′) = *F****a***(***x***′). Thus, *F*_***a***_(***x***) = *ct* = *F*_***a***_(***x***′). Secondly, it holds that ‖***x***‖_∞_ ≤ *β* because ***x*** is the solution of the TMO problem, and also ‖***x***′‖_∞_ ≤ *β* because (1) *γ* ≤ *β*/*nα* (by assumption) and (2) ‖***x***′‖_∞_ ≤ *n* × ‖*c*‖_∞_ × ‖***z***′‖_∞_ ≤ *nαγ* ≤ *β*. Lastly, we can see that ***x*** ≠ ***x***′. Note that in case 1 where ***z*** is computed as ***x****c*^−1^, we see that ***z*** ≠ ***z***′ because of the fact that ‖***z***‖_∞_ > *γ* (in case 1) and ‖***z***′‖_∞_ ≤ *γ* (in step 1). This inequality means that ***x****c*^−1^ ≠ ***x***′*c*^−1^, resulting in ***x*** ≠ ***x***′.

Next, in case 2, we show that there is another algorithm C that uses A to solve the OW problem of a GCK function. Given (***a***, *t*) as input, C does as follows:

1. Run A on input (***a***, *t*) and get (***x***, *c*) from A.
2. Output ***z*** = *c*^−1^***x*** as a solution.

The reason why ***z*** is a solution for the OW problem is that it holds that *F*_***a***_(***z***) = *t* and ‖***z***‖ ≤ *γ* by the condition of case 2.

As a result, the ability for A to solve the TMO problem is transferred to that of solving the CR or OW problem of a GCK function. We see that ${\text{Adv}}_{n,m,q,\alpha ,\beta }^{\text{TMO}(\text{case}\;1)}\leq {\text{Adv}}_{n,m,q,\beta }^{\text{CR}}$ and ${\text{Adv}}_{n,m,q,\alpha ,\beta }^{\text{TMO}(\text{case}\;2)}\leq {\text{Adv}}_{n,m,q,\gamma }^{\text{OW}}$ in case 2. This completes the proof.

#### 3.1.2 Extension to module GCK-TMO problem

The GCK function can be extended to a module setting where *F*_***A***_(***x***) = ***A*** ***x*** for $A\in R_{n,q}^{k\times \ell }$ and $x\in R_{\left[-\beta ,\beta \right]}^{\ell \times 1}$. Accordingly, the previous OW, CR, and TMO problems of GCK function can each be addressed with similarly defined problems in a module setting. In particular, we define a module version of TMO problem as below.

**Definition 7** (Module GCK-TMO Problem). *For integers* n,k,ℓ,q,α,β∈N, *the Module GCK-TMO problem is defined as follows: given* $A\in R_{n,q}^{k\times \ell }$ *and* $t\in R_{n,q}^{k\times 1}$, *find* $\left(x,c\right)\in R_{n,q}^{\ell \times 1}\times R_{n,q}$ *such that* ‖*c*‖_∞_ ≤ *α*, ‖***x***‖_∞_ ≤ *β satisfying*

$$\begin{array}{c} F_{A}\left(x\right)=ct. \end{array}$$ (3)

Similarly, we define ${\text{Adv}}_{n,k,\ell ,q,\alpha ,\beta }^{\text{M-TMO}}$ to be the advantage of an algorithm A solving the TMO problem of Module-GCK function over the ring *R*_*n*,*q*_. With two newly defined advantages ${\text{Adv}}_{n,k,\ell ,q,\beta }^{\text{M-CR}}$ and ${\text{Adv}}_{n,k,\ell ,q,\gamma }^{\text{M-OW}}$ (for some positive integer *γ*) regarding Module-GCK function, we can prove the following theorem.

**Theorem 2**. *Let n and ρ be power-of-2 integers such that n* ≥ *ρ*, *and q is a prime such that q* ≡ 2*ρ* + 1 (mod 4*ρ*). *For integers* k,ℓ,α,β,γ∈N *satisfying* $\alpha <\left(1/\sqrt{\rho }\right)\times q^{1/\rho }$, (2*β* + 1)^*nℓ*^ ≫ *q*^*nk*^ *and nαγ* ≤ *β*, *it holds that* ${\text{Adv}}_{n,k,\ell ,q,\alpha ,\beta }^{M-TMO}\leq {\text{Adv}}_{n,k,\ell ,q,\beta }^{M-CR}+{\text{Adv}}_{n,k,\ell ,q,\gamma }^{M-OW}$.

The proof can be done by the same argument as in the proof of Theorem 1 so we omit the proof in this paper.

### 3.2 Proposed GCKSign scheme

#### 3.2.1 Construction

For the security parameter λ, GCKSign generates the public parameters, *params*, as follows: choose an integer *n* such that *n* = 2^*a*^ for an integer a∈N (indeed, we set *n* = 256 for all parameter sets), and choose a prime modulus *q* and positive integers *k*, *ℓ*, *B*, *h*, *L*_*s*_ and *η*. Then, *params* is given by (*n*, *q*, *k*, *ℓ*, *B*, *h*, *L*_*s*_, *η*). Also, GCKSign requires a hash functions $H:{\left\{0,1\right\}}^{*}\to {\left\{0,1\right\}}^{\ell_{1}}$ and an encoding function $Encode:{\left\{0,1\right\}}^{\ell_{1}}\to R_{n,q}^{\left(h\right)}$. It is assumed that *params* and two functions (*H*, *Encode*) are used for all algorithms in GCKSign.

The key generation, signing, and verification algorithms of GCKSign are described as follows:

**KeyGen**. This algorithm first chooses random 256-bit seeds seed_***A***_ and seed_***s***_. It samples public polynomials ***A*** uniformly at random over $R_{n,q}^{k\times \ell }$ by expanding seed_***A***_ and secret polynomials *s*_1_, …, *s*_*ℓ*_ uniformly at random over *R*_[−*η*,*η*]_ by expanding seed_***s***_. Next, it computes a polynomial ***t*** = *F*_***A***_(***s***) = ***A*** ⋅ ***s***). Finally, it outputs a public key *pk* = (***t***, seed_***A***_) and a secret key *sk* = (***s***, seed_***A***_).

**Algorithm 1**: KeyGen

 **Input** :security parameter λ

 **Output**: public key *pk* = (***t***, seed_***A***_), and secret key *sk* = (***s***, seed_***A***_)

**1** seed_***A***_, seed_***s***_ ← {0, 1}^256^;

**2** $A\leftarrow {\text{sample}}_{A}\left({\text{seed}}_{A}\right)\in R_{n,q}^{k\times \ell }$ ;

**3** $s\leftarrow {\text{sample}}_{s}\left({\text{seed}}_{s}\right)\in R_{\left[-\eta ,\eta \right]}^{\ell \times 1}$ ;

**4** $t\leftarrow F_{A}\left(s\right)=A\cdot s\;\text{mod}\;q$ ;

**5** *pk* ← (***t***, seed_***A***_);

**6** *sk* ← (***s***, seed_***A***_);

**7 return** (*pk*, *sk*);

**Sign**. This algorithms first regenerates the public polynomials ***A*** from seed_***A***_. It chooses a random 256-bit seed seed_***y***_. It samples polynomials ***y*** uniformly at random over $R_{\left[-B,B\right]}^{\ell \times 1}$ by using seed_***y***_. Next, it computes v=A·ymodq. It obtains $\hat{c}$ by computing the hash function *H*(***v***, *μ*) together with the message *μ*. It obtains a sparse polynomial $c\in R_{n,q}^{\left(h\right)}$ by running $Encode(\hat{c})$ and computes ***z*** = ***y*** + *c* ⋅ ***s***. If $z\notin R_{\left[-B+L_{s},B-L_{s}\right]}^{\ell \times 1}$, then it goes to the step that samples *y* and repeats the subsequent steps. Finally, it outputs a signature $\sigma =(z,\hat{c})$.

**Algorithm 2**: Sign

 **Input** : message *μ*, and secret key *sk* = (***s***, seed_***A***_)

 **Output**: signature $\left(z,\hat{c}\right)$

**1** $A\leftarrow {\text{sample}}_{A}\left({\text{seed}}_{A}\right)\in R_{n,q}^{k\times \ell }$

**2** seed_***y***_ ← {0, 1}^256^;

**3** $y\leftarrow {\text{sample}}_{y}\left({\text{seed}}_{y}\right)\in R_{\left[-B,B\right]}^{\ell \times 1}$ ;

**4** v=A·ymodq ;

**5** $\hat{c}\leftarrow H\left(v,\mu \right)\in {\left\{0,1\right\}}^{\ell_{1}}$ ;

**6** $c\leftarrow Encode\left(\hat{c}\right)\in R_{n,q}^{\left(h\right)}$ ;

**7** ***z*** ← ***y*** + ***s*** ⋅ *c*;

**8** **if** $z\notin R_{\left[-B+L_{s},B-L_{s}\right]}^{\ell \times 1}$ **then**

**9**  goto step 2;

**10 end if**

**11 return** $\sigma =(z,\hat{c})$ ;

**Verify**. This algorithm first derives a polynomial c from $\hat{c}$ in the signature. It regenerates the public polynomials ***A*** from the seed seed_***A***_. Next, it computes v=A·z-c·tmodq. The value ***v*** is used to compute the hash value *H*(***v***, *μ*) together with the message *μ*. It accepts the signature if the hash value matches the signature $\hat{c}$ and $z\in R_{\left[-B+L_{s},B-L_{s}\right]}^{\ell \times 1}$.

**Algorithm 3**: Verify

 **Input** : messasge *μ*, signature $\sigma =(z,\hat{c})$, and public key *pk* = (***t***, seed_***A***_)

 **Output**: {1, 0} // accept or reject signature

**1** $c\leftarrow Encode\left(\hat{c}\right)\in R_{n,q}^{\left(h\right)}$ ;

**2** $A\leftarrow {\text{sample}}_{A}\left({\text{seed}}_{A}\right)\in R_{n,q}^{k\times \ell }$ ;

**3** v=A·z-t·cmodq ;

**4** **if** $z\notin R_{\left[-B+L_{s},B-L_{s}\right]}^{\ell \times 1}\vee \hat{c}\neq \text{H}\left(v,\mu \right)$ **then**

**5**  **return** 0;

**6 end if**

**7 return** 1;

#### 3.2.2 Correctness

The way that GCKSign works is almost the same as Schnorr signature scheme, except for (1) rejection sampling in the Sign algorithm and (2) the usage of the encoding function. For a correctly generated $\sigma =(z,\hat{c})$, the first condition such that ‖***z***‖_∞_ < *B* − *L*_*s*_ holds, because it is the same as in the Sign algorithm, and the second condition is also guaranteed by the following equation

$$\begin{array}{c} Az-tc=A(y+sc)-(As)c=Ay. \end{array}$$ (4)

### 3.3 Security proof

**Theorem 3**. *Assume that* ‖*c****s***‖_∞_ ≤ *L*_*s*_ *and H is modeled as a random oracle. GCKSign is UF-CMA secure in the random oracle model if the TMO problem of the Module-GCK function is hard. That is, for any PPT adversary* A *with* ${\text{Adv}}_{DS}^{UF-CMA}\left(A\right)$, *making at most q*_*h*_ *hash queries and at most q*_*s*_ *signature queries, there exists a PPT algorithm* B *that solves the TMO problem with* ${\text{Adv}}_{n,k,\ell ,q,2,2(B-L_{s})}^{TMO}$, *where*

$$\begin{array}{c} {\text{Adv}}_{DS}^{UF-CMA}\left(A\right)\leq \frac{q_{h}q_{s}}{{\left(2B+1\right)}^{n\ell }}+\sqrt{q_{h}{\text{Adv}}_{n,k,\ell ,q,2,2(B-L_{s})}^{TMO}}+\frac{q_{h}}{2^{\ell_{1}}}. \end{array}$$ (5)

*Proof*. In order to prove the security of GCKSign, we define a sequence of hybrid games **G**_0_, **G**_1_, **G**_2_, where **G**_0_ is the original UF-CMA security game defined in Chapter 2.2 and **G**_2_ is the final game in which the success probability of A can be easily bounded by the hardness of the TMO problem. For each game **G**_*i*_, we define an event *δ*_*i*_ where A successfully outputs a forgery in the game **G**_*i*_.

**Game G**_0_. In this game, a challenger C runs the key generation algorithm to get (*pk*, *sk*) and gives *pk* to A. Whenever A asks a hash query (***v***, *μ*), C gives the same answer to A if the query has been asked before. If not, C chooses a random $\hat{c}\in {\left\{0,1\right\}}^{\ell_{1}}$ and gives it to A. Whenever A asks a signature query *μ*, C runs the signing algorithm to get a signature *σ* and gives $\sigma =(z,\hat{c})$ to A. Finally, A outputs (μ*, σ*) with the condition that μ* was not queried. C returns Verify(*pk*, μ*, σ*) as the output of the experiment. Thus, we get $\text{Pr}\left[\delta_{0}\right]={\text{Adv}}_{\text{PKS}}^{\text{UF-CMA}}\left(A\right)$.

**Game G**_1_. **G**_1_ is the same as **G**_0_ except that the signing queries are replaced by the MidSign algorithm (see Algorithm 4 below). Since two games **G**_0_ and **G**_1_ are the same except that the random hash value $\hat{c}$ is programmed before receiving the input (*v*, *μ*). A can not tell if the signing oracle was answered by the Sign algorithm or the MidSign algorithm.

**Algorithm 4**: MidSign

 **Input** : message *μ*, public key (***A***, ***t***)

 **Output**: signature $\left(z,\hat{c}\right)$

**1** Choose ***y*** uniformly at random from $R_{\left[-B,B\right]}^{\ell \times 1}$;

**2** Choose $\hat{c}\in R_{n,q}^{\left(h\right)}$ uniformly at random;

**3** Compute $c=Encode(\hat{c})$;

**4** Compute ***z*** = ***y*** + *c****s***. If ‖***z***‖_∞_ > *B* − *L*_*s*_, then

**5**  retry at step 1;

**6** Compute ***v*** = ***Ay***;

**7** Re-program the hash oracle *H*(⋅) so that

**8** $H\left(v,\mu \right)=\hat{c}$ ;

**9** Return $\sigma =(z,\hat{c})$;

Let *inconsistency* be the event that any $\hat{c}$ is previously assigned to an input value (***v***, *μ*) queried by A. This event occurs when ***v*** (computed by C) becomes one of values queried by A, assuming that *μ* is the same message. Since $v=Ay\in R_{q}^{k\times 1}$ and ***y*** is chosen uniformly at random from $R_{\left[-B,B\right]}^{\ell \times 1}$, the total number of ***v*** is (2*B* + 1)^*nℓ*^. For *q*_*h*_ number of hash queries, the probability that any $\hat{c}$ becomes one of queried ***v*** values is at most *q*_*h*_/(2*B* + 1)^*nℓ*^. Also, since A issues at most *q*_*s*_ number of signature queries, the probability that *inconsistency* happens is at most *q*_*h*_*q*_*s*_/(2*B* + 1)^*nℓ*^. Obviously, unless *inconsistency* happens, **G**_1_ is the same as **G**_0_. Thus, we see that |Pr[*δ*_1_] − Pr[*δ*_0_]| ≤ *q*_*h*_*q*_*s*_/(2*B* + 1)^*nℓ*^.

**Game G**_2_. **G**_2_ is the same as **G**_1_ except that the signature queries are replaced by the SimSign algorithm (see Algorithm 5 below). **G**_2_ is the same as **G**_1_ except the way that ***z*** and ***v*** are generated. Though $\sigma =(z,\hat{c})$ is generated in **G**_2_ without using *sk* = ***s***, it is infeasible for A to differentiate between **G**_1_ and **G**_2_ because of the zero-knowledge property of the relevant identification protocol.

**Algorithm 5**: SimSign

 **Input** : message *μ*, public key (***A***, ***t***)

 **Output**: signature $\left(z,\hat{c}\right)$

**1** Choose ***z*** uniformly at random from $R_{\left[-B+L_{s},B-L_{s}\right]}^{\ell \times 1}$;

**2** Choose $\hat{c}\in R_{n,q}^{\left(h\right)}$ uniformly at random;

**3** Compute $c=Encode(\hat{c})$;

**4** Compute ***v*** = ***Az*** − *c****t***;

**5** Re-program the hash oracle *H*(⋅) so that

**6** $H\left(v,\mu \right)=\hat{c}$ ;

**7** Return $\sigma =(z,\hat{c})$;

The remaining part is to check if A can distinguish between the two distributions of $\left(z,\hat{c},v\right)$ in **G**_1_ and **G**_2_, especially in terms of ***v***. In **G**_1_, ***v*** is computed as ***v*** = ***Ay*** for some $y\in R_{\left[-B,B\right]}^{\ell \times 1}$, whereas in **G**_2_ ***v*** is computed as ***v*** = ***Az*** − *c****t*** for some $z\in R_{\left[-B+L_{s},B-L_{s}\right]}^{\ell \times 1}$. what needs to be checked here is that ***v*** = ***Az*** − *c****t*** in **G**_2_ can be expressed as ***v*** = ***A*r** for some (unknown) $r\in R_{\left[-B,B\right]}^{\ell \times 1}$. If so, the two distribution of $\left(z,\hat{c},v\right)$ in **G**_1_ and **G**_2_ are identical from the A’s point of view. To guarantee this, we can set ***r*** = ***z*** − *c****s*** for some (unknown) secret key $s\in R_{\left[-\eta ,\eta \right]}^{\ell \times 1}$ such that ***t*** = ***As***. Then, we see that ***Ar*** = ***A***(***z*** − *c****s***) = ***Az*** − *c****t*** = ***v***. Since ‖*c****s***‖_∞_ ≤ *L*_*s*_ (by assumption) and ‖***z***‖_∞_ ≤ *B* − *L*_*s*_ (by choosing ***z***), we get ‖***r***‖_∞_ ≤ ‖***z***‖_∞_ + ‖*c****s***‖_∞_ ≤ *B* − *L*_*s*_ + *L*_*s*_ = *B*, which is the same distribution as **G**_1_. As a result, we have that Pr[*δ*_2_] = Pr[*δ*_1_].

We now apply the generalized forking lemma of Bellare and Neven [6] to **G**_2_. With the probability at least $\epsilon^{\prime }(\epsilon^{\prime }/q_{h}-1/2^{\ell_{1}})$ where Pr[*δ*_2_] = *ϵ*′, we obtain two signatures $\left(z,\hat{c}\right)$ and $\left(z^{\prime },{\hat{c}}^{\prime }\right)$ for $\hat{c}\neq {\hat{c}}^{\prime }$ such that

$$Az-ct=v=Az^{\prime }-c^{\prime }t.$$

C then simply sets ***x*** = ***z*** ‒ ***z***′, $\tilde{c}=c-c^{\prime }$, and outputs $\left(x,\tilde{c}\right)$ as the solution of the TMO problem, satisfying $Ax=\tilde{c}t$. We can check that ‖***x***‖_∞_ ≤ 2(*B* − *L*_*s*_) and $\|\tilde{c}{\|}_{\infty }\leq 2$. Thus, we have that $\epsilon^{\prime }(\epsilon^{\prime }/q_{h}-1/2^{\ell_{1}})\leq {\text{Adv}}_{n,m,q,2,2(B-L_{s})}^{\text{TMO}}$, which is further simplified as $\epsilon^{\prime }\leq \sqrt{q_{h}{\text{Adv}}_{n,m,q,2,2(B-L_{s})}^{\text{TMO}}}+q_{h}/2^{\ell_{1}}$. This completes the proof.

### 3.4 Parameter settings

Table 1 presents several conditions necessary for choosing the parameters for GCKSign. The proof of Theorem 3 indicates that the TMO problem of the Module-GCK function is specified by the parameters as *α* = 2 and *β* = 2(*B* − *L*_*s*_). Also, because the number of non-zero coefficients of *c* is *h*, for any $s\in R_{\left[-\eta ,\eta \right]}^{\ell \times 1}$, it holds that ‖*c****s***‖_∞_ ≤ *hη*, allowing for setting *L*_*s*_ = *hη*. Based on these requirements, Table 2 proposes three sets of parameters for GCKSign. We set *η* = 1 as the bound for the secret key polynomial ***s***, and set *ρ* = 8 for all parameter sets in order to choose *q* that meets the requirement (2) in Table 1.

**Table 1:** Requirements for parameter selection.

	Parameter Requirements
(1)	*n* = 2^*a*^ for a∈N
(2)	*q* ≡ 2*ρ* + 1 (mod 4*ρ*), *ρ* | *n* and $\alpha \leq \left(1/\sqrt{\rho }\right)\times q^{1/\rho }$
(3)	*nαγ* ≤ *β*
(4)	2h×(nh)≥2λ
(5)	‖*c****s***‖_∞_ ≤ *L*_*s*_
(6)	(2*β* + 1)^*nℓ*^ ≫ *q*^*nk*^

**Table 2:** Parameter sets for GCKSign.

	1	2	3
*n*	256	256	256
*q*	2^25^ − 463	2^26^ − 111	2^27^ − 79
(*k*, *ℓ*)	(2,5)	(3,8)	(7,17)
*h*	24	39	74
2h(nh)	135	192	291
*B*	2^15^ − 1	2^15^ + 2^9^ − 1	2^18^ −1
*η*	1	1	1
*L*_*s*_	24	39	74
# of RS	2.55	3.38	3.41
LWE Hardness (Core-SVP)			
BKZ (*b*)	245	476	1047
Classical	71	139	306
Quantum	64	126	277
SIS Hardness (Core-SVP)			
BKZ (*b*)	251	459	996
Classical	73	134	291
Quantum	66	121	264
Performance (K cycle)			
KeyGen	361	796	4,276
Sign	839	4,185	8,823
Verify	413	886	4,474

# of RS: number of rejection sampling. *b*: BKZ block size.

### 3.5 Concrete security analysis

As shown above, the security of GCKSign relies on the TMO problem of Module-GCK function relative to ${\text{Adv}}_{n,k,\ell ,q,2,2(B-L_{s})}^{\text{TMO}}$. By Theorem 2, the security is further reduced to the CR and OW problems of Module-GCK function relative to ${\text{Adv}}_{n,k,\ell ,q,2(B-L_{s})}^{\text{M-CR}}$ and ${\text{Adv}}_{n,k,\ell ,q,\gamma }^{\text{M-OW}}$ where *γ* is determined by the requirement (3) *γ* ≤ *β*/(*nα*) in Table 1. Also, we use the fact the CR problem of Module-GCK function is reduced to Module-SIS problem relative to ${\text{Adv}}_{n,k,\ell ,q,2(B-L_{s})}^{\text{M-SIS}}$, and (using the Hermite normal form) the OW problem of Module-GCK function is reduced to Module-LWE problem relative to ${\text{Adv}}_{n,k,(\ell -k),q,\gamma }^{\text{M-LWE}}$. Strictly speaking, 4(*B* − *L*_*s*_) is more correct than 2(*B* − *L*_*s*_), but we follow the same analysis as Dilithium [11] to analyze the security of Module-SIS. Regarding the choice of *γ*, especially, *γ* can be chosen from any positive integer satisfying *γ* ≤ 2(*B* − *L*_*s*_)/(2^8^ × 2), but we set *γ* = 1 for the Module-LWE problem. This is because a smaller ratio of *q*/*γ* (i.e., modulus-to-noise) generally provides stronger concrete security against known lattice attacks [15], and thus decreasing *γ* is more advantageous for an adversary solving Module-LWE problem when *q* is fixed. Eventually, the concrete security of GCKSign is estimated by the best-known lattice attacks against Module- {SIS, LWE} problems relative to ${\text{Adv}}_{n,k,\ell ,q,2(B-L_{s})}^{\text{M-SIS}}$ and ${\text{Adv}}_{n,k,(\ell -k),q,1}^{\text{M-LWE}}$, respectively. The existence of a solution with respect to *γ* = 1 is guaranteed by the key generation such that ***t*** = ***As*** with ***A*** ∈ *R*^*k*×*ℓ*^ and $s\in R_{\left[-1,1\right]}^{\ell \times 1}$.

To analyze the concrete security of the above Module- {SIS, LWE} problems, we use the BKZ lattice reduction algorithm [16] as the best-known lattice attacks. There are a variety of approaches to measure the running time of BKZ [16–18]. In general, an SVP (Shortest Vector Problem) solver is the main building block of the BKZ algorithm. Regarding the number of SVP oracle calls that the BKZ algorithm makes, the Core-SVP model [18] assumes that an SVP oracle is required only once in a conservative model. The best known classical SVP solver runs in time ≈ 2^0.292×*b*^ and the best known quantum SVP solver runs in time ≈ 2^0.265×*b*^. Therefore, we decide to adopt the BKZ cost model of 0.292*b* for the classical model and the BKZ cost model of 0.265*b* for the quantum model where *b* is the BKZ block size. Table 2 shows the concrete security level of GCKSign, according to each parameter set. To estimate the hardness of the parametrized Module- {SIS, LWE} problems, we make use of the SIS and LWE estimators in [11] that reflect the above-mentioned BKZ algorithm.

## 4 Discussion

### 4.1 Comparison

Table 3 presents the comparison between the two GCK-based signatures, [4] and GCKSign. In terms of security, [4] is based on the collision-resistance of GCK function, whereas GCKSign is based on the TMO problem of Module-GCK function. Because of the collision resistance in [4], their GCK function must be set to guarantee the WI property, which requires to satisfy the condition (at least) (2*η* + 1)^*nℓ*^ ≥ *q*^*nk*^ × 2^128^ for the coefficient bound *η* of a secret key polynomial. [4] sets *η* = 2047, which turns out to increase the sizes of public/secret keys and signatures. On the other hand, GCKSign removes the WI property by proving their security under the TMO problem of GCK function, and importantly is able to relax to the condition (at least) (2*β* + 1)^*nℓ*^ ≥ *q*^*nk*^ × 2^128^ for *β* = 2(*B* − *L*_*s*_). Because of this relaxation, GCKSign can set much smaller values of *q* and *η*, and obtain a significant increase in efficiency compared to [4]. For instance, Table 3 shows that at the (almost) same 132-bit security level, the signature size of GCKSign is about 3.4 (≈ 14875/4384) times shorter and the public-key size of GCKSign is about 2.4 (≈ 6125/2528) times shorter, compared to [4].

**Table 3:** Comparison between GCK-based signatures.

	*n*	(*k*, *ℓ*)	*q*	*η*	*sig* (bytes)	*pk* (bytes)	*pk* + *sig* (bytes)	Classical security	Hardness problem
[4]	512	(1, 5)	≈ 2^60^	2047	9, 000	3, 875	12, 875	71	GCK-CR
	512	(1, 8)	≈ 2^96^	2047	14, 875	6, 125	21, 000	132	
	1, 024	(1, 8)	≈ 2^96^	2047	30, 750	12, 250	43, 000	283	
[11]	256	(4, 4)	≈ 2^23^	2	2, 420	1, 312	3, 732	123	Module-SIS, Module-LWE
	256	(6, 5)	≈ 2^23^	4	3, 293	1, 952	5, 245	182	
	256	(8, 7)	≈ 2^23^	2	4, 595	2, 592	7, 187	252	
Ours	256	(2, 5)	≈ 2^25^	1	2, 592	1, 632	4, 224	71	Module-SIS, Module GCK-TMO
	256	(3, 8)	≈ 2^26^	1	4, 384	2, 528	6, 912	134	
	256	(7, 17)	≈ 2^27^	1	10, 368	6, 080	16, 448	291	

However, the sizes of public key and signature of GCKSign are about 1.8 (≈ 2528/1312) times and 1.9 (≈ 4384/2420) times larger, compared to [11] at the (almost) same 132-bit security level. This is caused by the key recovery attack. An adversary may try to recover the secret key ***s*** from the public key *pk* = (***A***, ***t***), where ***t*** = ***As*** in case of GCKSign. Because ***s*** is sampled from $R_{\left[-\eta ,\eta \right]}^{\ell \times 1}$ and *η* = 1, this amounts to solving the OW problem of Module-GCK function, which is reduced to the Module-LWE problem relative to ${\text{Adv}}_{n,k,(\ell -k),q,1}^{\text{M-LWE}}$, and the dimension is reduced to *ℓ* − *k* instead of *ℓ* itself. Therefore, We need to increase the dimension for our scheme by *k* to maintain the security level which the LWE-based signature sets like Dilithium [11], and this leads to increase of the sizes of public key and signature at the end.

### 4.2 Performance analysis

In Table 2, we evaluate the performance of our implementations on a 3.7GHz Intel Core i7-8700k running Ubuntu 20.04 LTS. The table shows the key generation, signing, and verification algorithms of our scheme. Our implementation codes are publicly available at https://github.com/KU-Cryptographic-Protocol-Lab/GCKS.

A polynomial multiplication is the most costly operation in implementing a cryptosystem. We choose the modulus *q* satisfying the condition *q* ≡ 2*ρ* + 1 mod 4*ρ* for some *ρ*|*n* to satisfy the security requirement. As a result, we can not use the “fully-splitting” NTT algorithm for the multiplication operation. Instead, we choose to follow the approach of [14], using “partially-splitting” NTT algorithm with a Karatsuba multiplication [19] and Toom-Cook polynomial multiplication method [20] to efficiently multiply in a partially-splitting ring.

## 5 Conclusion

In this paper, we have addressed the challenge of proving the security of GCK-based signature schemes without relying on the WI property. By introducing the TMO problem and showing its reduction to the one-wayness and collision-resistance problems of the GCK function, we have provided a solution to overcome the limitations imposed by the WI property in GCK-based signatures. Additionally, we have presented a more efficient GCK-based signature scheme, GCKSign, which extends the original scheme [4] by incorporating the Module-GCK function. Through the analysis of the Module GCK-TMO problem and its reduction to Module-{SIS, LWE} problems, we prove the security of GCKSign in the random oracle model without relying on the WI property.

One notable result of our work is the significant reduction in signature size and public key size compared to Lyubashevsky’s signature scheme [4]. GCKSign achieves approximately 3.4 times shorter signature size and 2.4 times shorter public key size at the same security level. This efficiency improvement directly stems from the elimination of the WI property.

Even though our scheme still has larger sizes than Dilithium [11], our findings not only contribute to the advancement of GCK-based signature scheme [4] but also provide insights into the design and analysis of cryptographic primitives based on structured lattice problems. The TMO problem introduces a novel perspective on approximate one-wayness, and its connections to existing hardness assumptions offer new avenues for future research. In future work, it would be interesting to explore the applicability of the TMO problem in other cryptographic protocols and settings.
